# Suppression of Oral Sweet Sensations during Consumption of Sweet Food in Humans: Effects on Gastric Emptying Rate, Glycemic Response, Appetite, Food Satisfaction and Desire for Basic Tastes

**DOI:** 10.3390/nu12051249

**Published:** 2020-04-28

**Authors:** Naomi Kashima, Kanako Kimura, Natsumi Nishitani, Masako Yamaoka Endo, Yoshiyuki Fukuba, Hideaki Kashima

**Affiliations:** 1 Faculty of Health Sciences, Hiroshima Shudo University, 1-1-1 Ozuka-higashi, Asaminami-ku, Hiroshima 731-3195, Japan; 2 School of Health Sciences, Prefectural University of Hiroshima, 1-1-71 Ujina-higashi, Minami-ku, Hiroshima 734-8558, Japan

**Keywords:** sweet taste suppression, cephalic phase, gastric emptying, glycemic response, desire for sweet taste

## Abstract

Suppression of oral sweet sensation (OSS) acutely reduces intake of sweet-tasting food due to lower liking. However, little is known about other physiological responses during both the prandial and postprandial phase. Here, we explored the effects of *Gymnema sylvestre* (GS)-based suppression of OSS of several types of sweet-tasting food (muffin, sweet yogurt, banana) on gastric emptying, blood glucose (BG), plasma insulin (PI), appetite indices (hunger, fullness and prospective consumption), satisfaction and desire for tastes. Fifteen healthy subjects (22 ± 3 years, 9 women) took part in the study. Subjects rinsed their mouth with either GS solution or distilled water before eating the sweet-tasting food. Subjects felt decreased sweet taste intensity and reduced taste liking associated with GS rinsing after consuming each food, compared with rinsing with distilled water (*p* < 0.05). Gastric emptying, BG, PI and appetite indices during and after the prandial phase did not significantly change with GS rinsing compared to rinsing with distilled water (*p* > 0.05). Higher desire for sweet taste as well as lower satisfaction (*p* < 0.05) in the postprandial phase were observed with GS rinsing. These results suggest that the suppression of OSS does not affect gastric emptying, glycemic response and appetite during and after consumption of sweet-tasting food.

## 1. Introduction

The sensory experience of eating is an important determinant of food intake control [1]. Specifically, the flavor is a strong factor influencing food choice [2]. The basic taste qualities have been associated with the physiological and nutritional value of food [3]. For instance, the sweet taste indicates the presence of carbohydrates, a major energy source, which may explain why humans are inherently inclined to eat sweet-tasting food and why they consume it eagerly. From an evolutionary perspective, it could help to secure energy intake and thereby survival, but in today’s environment, this might be a disadvantage because of the risk of overconsumption and obesity. Actually, excessive consumption of sweet-tasting food is linked to increased energy intake, being one of the major contributors to the global rise in obesity [4,5]. Since the proportion of energy obtained from sweet-tasting food depends on desire or liking for sweet taste [6,7], it is logical to assume that reducing sweet sensations might prevent overeating with sweet-tasting food. In other words, if the individual’s liking for the taste of sweet food can be controlled, it may be possible to reduce the intake of energy from sweet-tasting food.

Indeed, some previous studies reported that consumption of sweet-tasting food (e.g., candy and milkshake) can be reduced by suppressing oral sweet sensation (OSS) with the gymnema acids (GA) or tablets containing GA [8,9]. GA are a mixture of triterpene glycosides isolated from the plant *Gymnema sylvestre* (GS), which selectively suppresses sweet taste sensations in humans. While this seems to be a result of suppression of sweet taste receptors activation in the oral cavity with consequently reduced food liking for sweet-tasting food intake, the exact mechanism behind GA effects is not completely clear. To our knowledge, Stice et al. [10] have reported that GA suppress the activation of the parts of the brain’s reward system during drinking a sweet milkshake. However, previous studies [8,9,10] did not evaluate other physiological processes closely related to food intake control during the prandial phase (e.g., gastric emptying) [11]. When oral sweet receptor activation is suppressed during eating sweet-tasting food, gastric emptying may be delayed. This is because we have actually found that suppression of OSS during ingestion of glucose has a role in decreasing the gastric emptying and slowing blood glucose (BG) and insulin responses [12,13]. Therefore, to elucidate the role of suppression of OSS in decreased sweet-tasting food consumption, we should examine various physiological responses both during and after the prandial phase of consuming a uniform meal in a laboratory-based basic science approach. 

Specifically, suppression of OSS might potentially modulate gastric emptying and glycemic response via inhibition of “cephalic phase responses” (CPRs). Namely, CPRs refer to various digestive and hormonal responses in the mouth, stomach and pancreas that are initiated by olfactory, visual and taste cues before food reaches the stomach [14,15]. In particular, taste stimuli elicit the greatest gastric acid secretion among the various cephalic-phase cues [16]. The CPRs are modulated by food palatability and/or type of taste [17,18,19,20,21]. Therefore, as suppression of OSS can strongly decrease the individual’s taste liking to sweet food, it might also affect CPRs, such as glycemic response (via modulation of insulin response) and other physiological responses (such as gastric emptying). The best characterized CPR is cephalic phase insulin release (CPIR), the release of a small amount of insulin in response to the presence of sweet taste stimulus in the oral cavity, which leads to attenuated postprandial hyperglycemia in humans, irrespective of BG levels [18,19,22,23]. GS is a plant able to selectively suppress the sweet taste sensation in humans [24,25]. We found that GS-dependent suppression of OSS during ingestion of pure glucose solution slowed gastric emptying rate and glycemic response [12,13]. However, it is unknown whether suppressing OSS during consumption of a real sweet food affects both sweet taste liking and CPRs. 

Therefore, the main objective of the present study was to investigate the effects of GS-based suppression of OSS during consumption of sweet-tasting foods (muffin, sweet yogurt and banana) on gastric emptying, blood glucose (BG), plasma insulin (PI), as well as on appetite indices (hunger, fullness and prospective food consumption). In addition, considering that reduced sweet taste perception during eating a sweet-tasting food might influence the postprandial satisfaction and incline individuals towards sweeter stimuli in the next meal [26], we also intended to explore the patients’ satisfaction and desire for sweet, sour and salty tastes after the prandial phase.

## 2. Materials and Methods 

### 2.1. Participants

The study protocol fully complied with the Declaration of Helsinki, and the study was approved by the Prefectural University of Hiroshima Ethics Committee (approval number: 17HH002). Prior to the beginning of the study, each participant provided written, informed consent for participation. Six healthy males and nine healthy females (mean age 22 ± 3 years; mean height 162 ± 11 cm; mean weight, 56 ± 9 kg; mean body mass index (BMI), 21.2 ± 2.4 kg/m^2^) were included in the study. To determine the total sample size required in this study, we compared gastric emptying index (i.e., Tmax_-calc_) between two groups with or without rinsing GS solution using G*Power software (version 3.1.9.2) [12]. We obtained an effect size of 1.24 for a critical t of 2.365 at α of 0.05 and power (1 − β) of 0.80. Based on that, the calculated total sample size was eight subjects. Thus, we achieved the sample size sufficiently large to ensure 80% statistical power of the study. The participants had no food allergies, gastrointestinal symptoms, or history of significant diseases such as cardiovascular disease (hypertension with average systolic blood pressure of 140 mmHg or higher, or average diastolic blood pressure of 90 mmHg or higher), and they were not taking any medication. The day before the experiment, subjects consumed a standardized meal (hashed rice) of 469 kcal (Ginza hayashi; Meiji, Tokyo, Japan. Satounogohan; Satosyokuhin, Niigata, Japan) at 19:30–20:00. They arrived in the laboratory at 09:00 AM the following day. They abstained from strenuous exercise and consumption of alcohol or caffeine for at least one day before visiting the laboratory. 

### 2.2. Study Protocol

**Figure 1:**
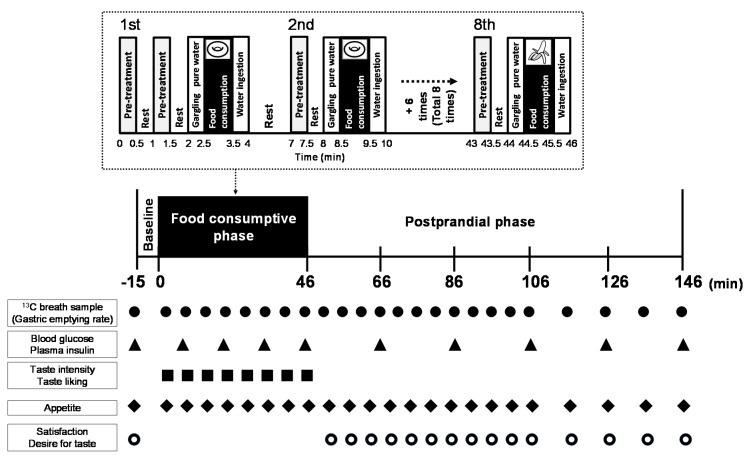
Scheme of the protocol of the study. Before consuming sweet-tasting food, participants rinsed their mouth for 0.5 min using 25 mL of distilled water or *Gymnema sylvestre* solution as pretreatment followed by a 0.5 min rest. These steps were repeated twice in the first step only. After that, the participants gargled with distilled water for 0.5 min, consumed a defined amount of sweet food for 1 min, and finally ingested water solution of ^13^C-sodium acetate for 0.5 min. The protocol was repeated eight times for a total duration of 46 min (food consumption, i.e., prandial phase): muffin (first four times, yogurt with added sucrose (next two times) and banana (last two times). In the postprandial phase, participants rested for 100 min (i.e., from 46th to 146th min) following consumption of sweet-tasting food. The figure also indicates the timing of measurements and evaluations of gastric emptying rate, plasma glucose and insulin, taste intensity and taste liking, hunger, fullness, prospective consumption, satisfaction and desire for taste.

The study had a randomized crossover design, and all participants came to the laboratory on two occasions (two trials). Namely, at each trial, participants were alternatively allocated to the control (n = 8) and treatment groups (n = 7). Female participants participated in both trials during the same phase of their menstrual cycles, because menstruation affects gastric emptying and blood glucose, insulin and glucagon-like peptide-1 concentrations [27]. In males, at least one week elapsed between the two trials. The day before the experiment, a distilled water with 100% GS powder (Kenkou Yasoucha, Yokohama, Japan) was boiled for 30 min and adjusted to 2.5% mass percent concentration (i.e., mass of solute (g)/mass of solution (g)) and then filtered. The filtered GS solution was used to suppress the sweet taste sensation in the oral cavity. Figure 1 presents the study protocol. The participants were seated on a chair in a quiet room with the temperature and humidity maintained at 25 ± 1 °C and 47 ± 7%, respectively. After participants were seated in a chair in a quiet resting room for 15 min to record the baseline value of all measurements, the pretreatment was done, in which participants rinsed their mouth for 30 s with 25 mL of either 2.5% GS solution (GS pretreatment) or distilled water (Control pretreatment), depending on the crossover group, followed by a 30 s rest. They were instructed not to swallow the pretreatment solutions. These steps were repeated twice. Then, the participants gargled with distilled water for 30 s until the rinsing solution was completely removed. They subsequently consumed a muffin (24 g) within 60 s and then ingested 50 mL of distilled water containing 12.5 mg of ^13^C-sodium acetate for 30 s to allow evaluation of gastric emptying rate using a ^13^C breath test. After resting for 180 s, the participants repeated the following steps seven times: rinsing their mouths with 25 mL of distilled water or 2.5% GS solution for 30 s, 30 s of rest, followed by gargling with distilled water for 30 s, and consumption of one of the sweet-tasting foods for 60 s. The order of successive consumption of sweet-tasting food was as follows: muffin (24 g, three times), 40 g of yogurt with 10 g of sucrose (two times), and 50 g of banana (two times). In total, each participant consumed 574 kcal (85.9 g of carbohydrates, 21.5 g of fats and 9.1 g of proteins) of sweet-tasting foods and 400 mL of distilled water containing 100 mg of ^13^C-sodium acetate and did not ingest pretreatment solutions (distilled water and GS solution). In this study, these sweet-tasting foods were assumed as a breakfast menu. In total, the prandial (food consumption) phase with eight episodes of ingesting sweet food lasted for 46 min, after which the participants rested on the seat for 100 min (postprandial phase). To maintain the suppressive level of OSS during consuming each sweet-tasting food, we repeatedly stimulated the oral cavity with pre-treatment (i.e., GS solution) just before consuming each sweet-tasting food. The concentrations of the pretreatment solutions were based on the findings from our previous studies [12,13]. This protocol has been designed to reliably suppress OSS and to consider the duration time of the oral stimulus [12,13]. All fluids (pretreatment solutions, water containing ^13^C sodium acetate and water used for gargling) were at a room temperature (25 ± 1 °C). 

### 2.3. Measurements

#### 2.3.1. Gastric Emptying Assessment

The rate of gastric emptying was evaluated using the ^13^C-sodium acetate breath test in accordance to our previous study [12,13]. Specifically, we dissolved 100 mg of ^13^C-sodium acetate in 400 g of distilled water. Participants ingested 50 mL of the solution containing 12.5 mg of ^13^C-sodium acetate at each of eight separate ingestion episodes. They were instructed to hold their breath for 10 s in order to obtain end-expiratory breath samples, which were collected in foil bags. Baseline breath samples were collected in large-capacity bags (PAYLORI-BAG5 L; Fukuda Denshi, Tokyo, Japan). Following the onset of the experiment, breath samples were collected in small-capacity bags (PAYLORI-BAG20; Fukuda Denshi) every six minutes during the prandial phase (from the 4th to 46th min), then during the postprandial phase every five minutes (between the 46th and 106th min) and every 10 min (from the 106th to 146th min) (Figure 1). ^13^CO_2_ enrichment in the breath samples was measured using an isotope ratio mass spectrometer (POCone; Otsuka Electronics, Hirakata, Japan). The time course of the percentage ^13^CO_2_ recovery per hour and the area under the curve (AUC) from the cumulative ^13^CO_2_ excretion from 0 to 146 min was determined by the trapezoidal method to evaluate gastric emptying rate in accordance to the previous study [13]. 

#### 2.3.2. Assessment of Perceived Taste Intensity, Taste Liking, Hunger, Fullness, Prospective Consumption, Satisfaction

Immediately after collecting the breath sample, the participants reported their perceived taste intensities of sweet, sour and salty as well as taste liking (Figure 1) using Japanese versions of the Labeled Magnitude Scale (LMS) [28] and Labeled Hedonic Scale (LHS) [29], respectively (Appendix A and Appendix B). The scale for LMS ranges between 0 and +100 mm, where a score of 0 mm reflects no sensation at all. The scale for LHS ranged between −100 mm (the most unpleasant taste imaginable) and +100 mm (the most pleasant taste imaginable). The characteristic feature of these scales is that the descriptions of perception are placed in vertical lines that are determined by estimations of their perceived magnitude. The ratings of taste intensity and liking were averaged per type of food.

In addition, each subject’s motivation to eat (i.e., appetite index) was assessed by measuring hunger, fullness and prospective consumption (Figure 1). The scales for hunger and prospective consumption ranged from 0 mm (most anorexigenic feeling) to +100 mm (most orexigenic feeling) (Appendix C). For fullness, the scale ranged from 0 mm (“not full at all”) to +100 mm (“very full”). In the postprandial phase, every subject rated his/her satisfaction as well as desire for sweet, sour and salty tastes (Figure 1) during 100 min in intervals similar to the breath test (Figure 1). The ratings of satisfaction ranged from 0 mm (“no satisfaction at all”) to +100 (“extreme satisfaction”). The desire for, for example, sweet taste, had a scale ranging from 0 mm (no desire at all for sweet food) to +100 mm (denoting strong desire for sweet food).

#### 2.3.3. Assessment of Blood Glucose and Insulin Levels

Capillary blood samples were collected at baseline as well as during the prandial phase (12th, 24th, 36th and 46th min) and the postprandial phase (66th, 86th, 106th, 126th and 146th min) (Figure 1) by pricking the index and middle fingers of the right hand. BG concentration was analyzed using a dedicated measurement device (Glucocard midea GT-1670; Arkray, Kyoto, Japan). Blood samples were collected into post-heparinized 75 µL capillary tubes and centrifuged at approximately 10,000–12,000× *g* for 5–6 min at room temperature (25 ± 1 °C) to obtain plasma samples. Plasma samples were refrigerated at −80 °C until measured. PI concentrations were measured using an enzyme immunoassay kit (Mercodia Insulin ELISA; Mercodia, Uppsala, Sweden).

### 2.4. Statistical Analysis

Data were presented as mean and standard error (SE) of the mean. The effects of time and pretreatment on the time course of pulmonary ^13^CO_2_ excretion rate, BG, PI, hunger, fullness, prospective food consumption, satisfaction and desire for specific taste were evaluated using two-way analysis of variance (ANOVA) for repeated measurements. If a significant main effect was detected, Dunnett’s and paired *t* post-hoc tests were conducted to determine the effects of time (change from baseline) and pretreatment (control versus GS conditions), respectively. The effect of treatment on subjective taste intensity and liking scores were evaluated using a two-way ANOVA for repeated measurements. If a significant main effect was detected, paired *t* post-hoc tests were conducted to determine the effect of pretreatment (control versus GS conditions). The gastric emptying index (i.e., AUCs of the percentage (^13^CO_2_ recovery)) was analyzed using the paired *t*-test between control and GS conditions. Statistical analyses were performed using SPSS version 18 (IBM Corp., Armonk, NY, USA). A *p*-value < 0.05 was considered significant.

## 3. Results

### 3.1. Subjective Taste Intensity and Taste Liking

**Figure 2:**
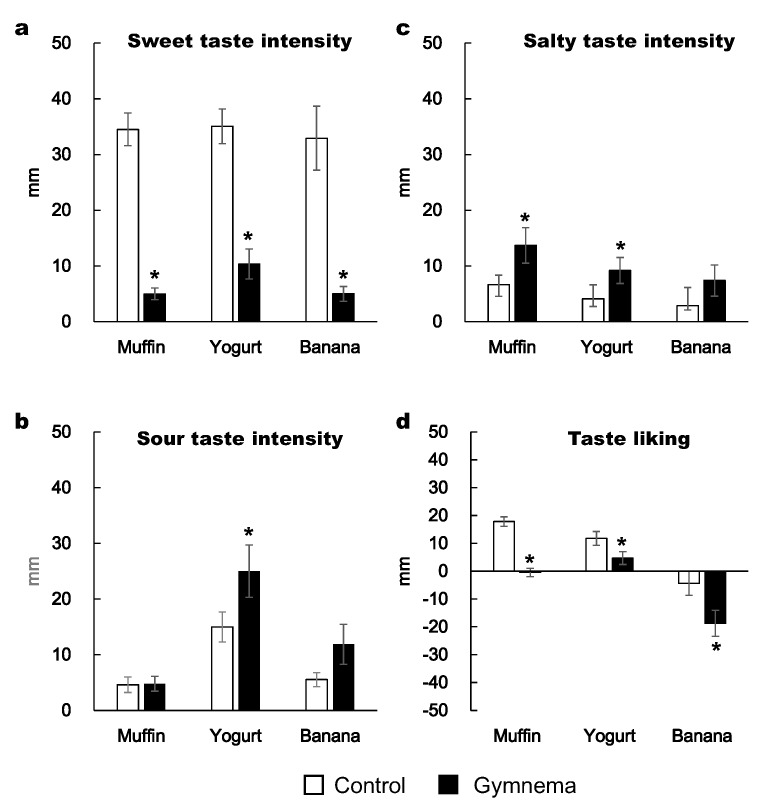
Subjective intensity of sweet (**a**), sour (**b**) and salty tastes (**c**), and taste liking (**d**) when consuming a muffin, yogurt and banana with *Gymnema sylvestre* (GS) pretreatment versus control pretreatment. Perceived taste intensity and liking were evaluated using the Labeled Magnitude Scale and Labeled Hedonic Scale, respectively. Mean ± standard error (SE). *: *p* < 0.05 between the two pretreatments.

Two-way repeated-measures ANOVA showed a significant effect of pretreatment on the subjective feeling of sweet taste intensity and taste liking scores. Namely, the subjective sweet taste intensities of muffin, yogurt and banana were substantially lower after GS mouth rinsing compared to the control pretreatment (*p* < 0.05; Figure 2a). The subjective sour taste intensity of yogurt was higher following GS pretreatment than in the control pretreatment (*p* < 0.05; Figure 2b). The subjective salty taste intensities of muffin and yogurt were higher at GS than in the control pretreatment (*p* < 0.05; Figure 2c). The food liking of muffin, yogurt and banana were substantially more reduced after the GS rinse than in the control rinse (*p* < 0.05; Figure 2d).

### 3.2. Gastric Emptying

**Figure 3:**
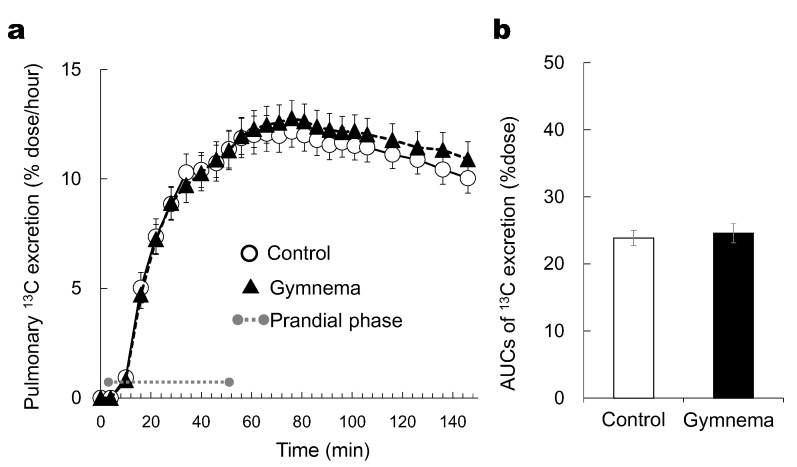
Time courses of ^13^C excretion responses (**a**) and the areas under the curve of ^13^C excretion rate for 146 min (**b**) during and after consuming sweet-tasting foods. Grey circles at both ends of dotted gray line denote the limits of prandial phase (consumption of sweet-tasting food). Mean ± SE.

We did not observe any significant effect of pretreatment (GS versus control) on the time course of the pulmonary ^13^CO_2_ excretion rate (*p* > 0.05) (Figure 3a). The AUCs of (^13^CO_2_) excretion rate was not significantly different between two treatment conditions (*p* > 0.05) (Figure 3b).

### 3.3. Blood Glucose and Plasma Insulin

**Figure 4:**
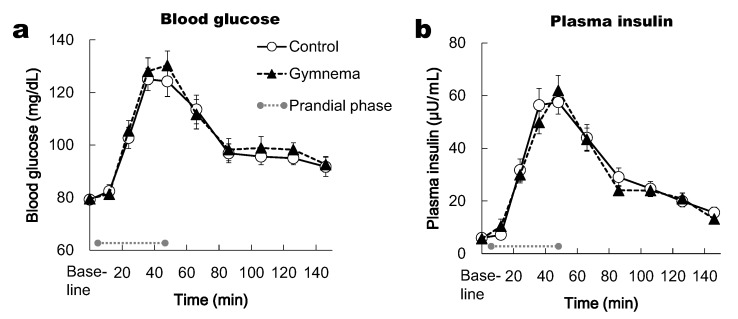
Time courses of blood glucose (**a**) and plasma insulin (**b**) responses during prandial and postprandial phases. Mean ± SE. Grey circles at both ends of the dotted gray line denote the limits of the prandial phase (consumption of sweet-tasting food).

The two-way repeated-measures ANOVA did not show a significant main of time for BG and PI responses (*p* < 0.05), however no effects of pre-treatment and the time × pre-treatment interaction were observed (*p* > 0.05). BG in the prandial and postprandial phase (in the period between the 24th and 126th min) was significantly higher from the baseline in both the control and GS pretreatment (*p* < 0.05) (Figure 4a). Likewise, PI in the prandial and postprandial phase (in the period between the 24th and 126th min) was significantly higher from the baseline in both the control and GS pretreatment (*p* < 0.05) (Figure 4b). In each time point, the BG and PI responses did not differ significantly between two pretreatments (Figure 4a,b).

### 3.4. Subjective Scores of Hunger, Fullness, Prospective Food Consumption, Satisfaction and Desire for Tastes

**Figure 5:**
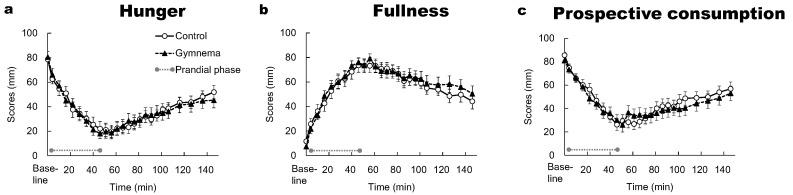
Time courses of hunger (**a**), fullness (**b**) and prospective consumption (**c**) during and after consuming sweet-tasting foods. Mean ± SE. Grey circles at both ends of the dotted gray line denote the limits of the prandial phase (consumption of sweet-tasting food).

The two-way repeated-measures ANOVA did not show a significant main effect of time on the ratings of hunger, fullness and prospective consumption (*p* > 0.05); likewise, no effects of pre-treatment and the time × pre-treatment interaction were observed (*p* > 0.05). After the 4th min of the study, the ratings of hunger and prospective consumption were significantly lower from the baseline in both the control and GS pretreatment (*p* < 0.05) (Figure 5a,c), whereas the ratings of fullness showed the opposite trend (*p* < 0.05) (Figure 5b). Following consuming all sweet-tasting food, the hunger, fullness and prospective consumption slowly started to return toward the baseline level in the control and GS pretreatment. At 146 min, neither of the appetite indices returned to baseline level in the control and GS pretreatment. In each time point, the ratings of hunger, fullness and prospective consumption did not significantly differ between the two pretreatments (*p* > 0.05).

**Figure 6:**
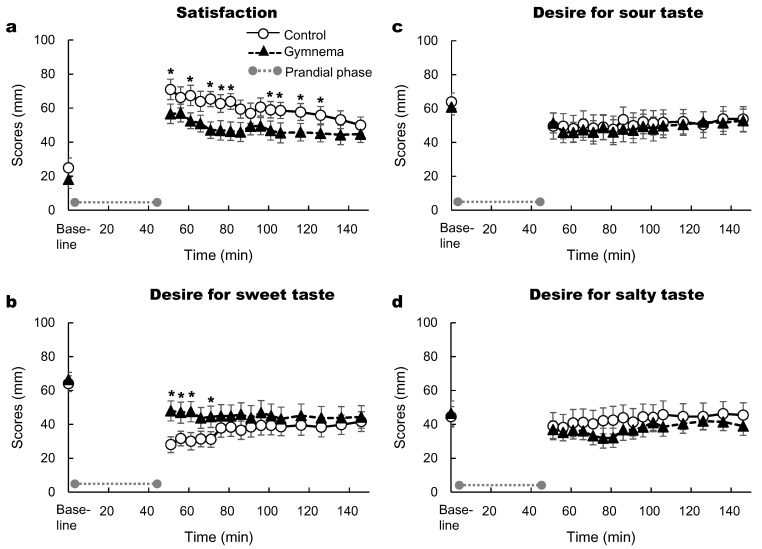
Time courses of satisfaction (**a**) and desire for sweet (**b**), sour (**c**) and salty (**d**) tastes after consuming sweet-tasting foods. Mean ± SE. Grey circles at both ends of the dotted gray line denote the limits of the prandial phase (consumption of sweet-tasting food). *: *p* < 0.05 between the two pretreatments.

The two-way repeated measures ANOVA showed significant effects of time and pretreatment on satisfaction (*p* < 0.05; Figure 6a), whereas no significant effect of the time × pre-treatment interaction was observed. Namely, the ratings of satisfaction were lower after the GS pretreatment. The two-way repeated measures ANOVA showed significant effects of time and the time × pre-treatment interaction on desire for sweet taste (*p* < 0.05; Figure 6b), however no effect of pre-treatment was observed. The ratings of desire for sweet taste were higher in the GS treatment than control (Figure 6b). There was a significant effect of time on sour and salty tastes, whereas no significant effects of time and the time × pre-treatment interaction were observed (*p* > 0.05; Figure 6c,d).

## 4. Discussion

We examined whether prandial and postprandial gastric emptying rate, BG, PI, hunger, satisfaction and desire for basic tastes are altered by the suppression (not abolishment) of OSS from a general sweet-tasting food. As expected, in the GS pretreatment, the ratings of subjective sweet taste intensity were strongly suppressed, which was enough to decrease the subject’s perceived taste liking. Under the conditions of suppression of taste receptor activation with antagonists (i.e., GS solution), the present study makes two main points. First, when real sweet-tasting food and the same amount of sweet-tasting food is consumed, the suppression (not abolishment) of OSS with GS decreased the liking derived from sweet-tasting food but did not change gastric emptying, glycemic responses and appetite. Second, during the postprandial phase, higher scores of desire for sweet taste and lower satisfaction were observed in GS compared with control pretreatment.

Gastric emptying is among the key factors for determining appetite [11] and postprandial blood glucose excursion [30]. For example, slowing the gastric emptying rate can attenuate postprandial glucose excursion and appetite, leading to mitigation of overeating and postprandial hyperglycemia. However, in the current experimental setting, we did not find that suppression of OSS by GS affected gastric emptying. We previously reported that suppression of OSS with GS during ingestion of glucose solution had a role in decreasing gastric emptying [12,13]. Inui-Yamamoto et al. [31] also reported that the gastric emptying response to ingestion of foods containing sweet-tasting substances is faster than that of non-sweet-tasting and bitter tasting substances. However, when such different tasting foods were infused into the stomach directly (i.e., bypassing the activation of taste receptors in the oral cavity), gastric emptying did not differ among three types of taste (i.e., tasteless, sweet taste and bitter taste foods) [31]. Thus, oral gustatory information might be associated with modulation of gastric emptying and, in particular, a sweet taste seems to be a unique and important cephalic stimulus for gastric motility. Thus, there seems to be some reasons for the lack of changes in gastric emptying with the suppression of OSS in this study. Modulation of gastric emptying rate associated with CPRs might depend on multiple factors, such as type of taste and its intensity and taste liking. In the GS pretreatment, perceived OSS could not be completely suppressed. We previously reported that the modified sham feeding (MSF) with 4% glucose solution increases blood flow in the celiac artery, which supplies the blood to the stomach, spleen, liver and pancreas in humans [32]. The mean score of sweet taste intensity with 4% glucose was found to be between weak and moderate ratings using LMS (i.e., in the same manner as the present study) [32]. Thus, even with the weak OSS, the gastric emptying might be accelerated. In addition, the GS pretreatment increased perceived sour and salty taste intensities compared with control. Sour tastes are associated with rotten foods, so we generally tend to avoid these foods. Salty-tasting foods signal the presence of sodium which is essential for maintaining the body fluid balance and blood circulation, while cautioning against the ingestion of excess salt [2]. There is no evidence on any relationship between sour and salty tastes and gastric emptying, whereas oral sour taste (but not sweet taste) stimulus decreases gastric myoelectrical activity in humans [33]. Some previous studies demonstrated that unpleasant taste (bitter taste or unappetizing food) decreases gastric motility and gastric emptying [20,21]. The GS pretreatment was associated with a lower taste liking compared to control, but it was not so unpleasant as to affect gastric emptying. To further unravel the detailed mechanisms of gastric emptying depending on complex tastes, we need to investigate the effects of MSF with type of taste and its intensity and liking on gastric emptying. In addition, the total time of direct oral stimulation by sweet-tasting foods (8 min) may have been too short to modulate gastric emptying, resulting in a lack of differences in gastric emptying between control and the GS pretreatment. Therefore, further studies should investigate longer time of direct oral stimulation by sweet-tasting foods to further elucidate the effects of OSS suppression on gastric emptying.

Whereas subjects’ perceived OSS and taste liking were suppressed by GS solution during consumption of sweet-tasting foods, the time courses of BG and PI responses were almost equivalent to the control trial. This might be because perceived OSS could not be completely abolished by GS as described above. MSF with OSS can slightly increase insulin secretion (i.e., CPIR) [18,19,22,23]. CPIR partially plays a role in suppressing postprandial glycemic response in humans. BG and PI responses did not differ between GS and control pretreatments in our study. Thus, suppression (not abolishment) of OSS using GS solution seems to reduce the liking derived from sweet-tasting food intake without affecting glycemic control.

The influence of OSS on appetite indices was not shown in this study in prandial and postprandial phases. The specific role of sweet taste in appetite regulation has been controversial [34]. Some studies reported that sweet taste itself can stimulate hunger [35,36,37,38], whereas some previous studies failed to show an effect of sweet taste on appetite and food intake in subsequent meals [39,40,41,42,43]. The latter seem to be supported by our results and accorded to the results of gastric emptying. However, if the individual would not use GS in the next meal, there are concerns that they would overeat with sweet-tasting foods. Indeed, this is because lower postprandial satisfaction and higher desire for sweet taste were observed in GS compared with those in the control condition. Noel et al. [26] demonstrated that GS-dependent reduced peripheral gustatory input (from taste receptors activated by sweeteners) predisposes to desire for sweeter stimuli. It is important to determine whether the discrepancy between appetite and liking affects food intake control. Future research will be needed to investigate the effect of suppression of OSS in the first meal on food intake and its selection in the second meal.

Our study has some limitations. First, in this study, all participants had no specific training to evaluate taste sensations. Therefore, it is difficult to determine whether they could distinguish fine taste differences and each of the basic taste attributes. Second, the time period following consumption of the sweet-tasting food was relatively short, and not all variables returned to baseline values. We only documented the effect of OSS with sweet-tasting foods on CPRs for 100 min during the postprandial phase. We focused on physiologic responses during the postprandial initial phase based on our previous work. However, from the perspective of clinical implication, the effect of CPRs on glucose tolerance is of special interest. During an oral glucose tolerance test, BG and PI would normally be monitored for 120 min following ingestion of the glucose drink. Third, sweet taste solutions containing ^13^C-sodium acetate were orally administrated in eight divided doses for 42 min, but there was no bolus dose. This was done to simulate standard, non-experimental conditions. Thus, the present data of gastric emptying rate should not be compared to other studies which use a standardized ^13^C breath test.

In this study, we focused on the psychological and physiological responses to suppression of OSS using a realistic diet. We believe that our findings set the basis for further research to address both basic mechanisms and practical applications. Future studies will need to further investigate the effects of the first meal with OSS on the subsequent (e.g., next meal) food intake without OSS and the extent of OSS that effectively reduces food intake of sweet-tasting food (i.e., dose-response behavior of OSS). Moreover, in a situation where a less sweet-tasting meal (e.g., meat and fish dishes, grains (bread and rice), salad, etc.) and a sweet-tasting food are simultaneously served, it is necessary to determine whether the intake of sweet-tasting food is reduced by OSS suppression; in that case, we should also determine whether there is compensatory increase in the intake of non-sweet-tasting food. Finally, acute, short-term and long-term interventional studies are needed that not only focus on healthy humans but also on obese and individuals with type two diabetes who need to manage their overeating and/or postprandial hyperglycemia derived from carbohydrate-rich foods.

## 5. Conclusions

In summary, we used GS to cause the suppression of OSS of sweet-tasting foods and evaluate its effects on gastric emptying rate, glycemic responses, hunger, fullness, prospective food consumption and desire for basic tastes both during and after the prandial phase. Suppression (not abolishment) of OSS during consumption of a general sweet-tasting food does not affect gastric emptying and glycemic responses as CPRs, whereas it decreases postprandial satisfaction and acutely changes the individual’s desire for sweet taste.

## Appendix A

**Table A1:** Rating of sweet, sour and salty taste intensities of sweet-tasting foods (muffin, sweet yogurt and banana).

English	Japanese
No sensation	何も感じない
Barely noticeable	最低限感じられる
Weak	弱い
Moderate	中等度の
Strong	強い
Very strong	とても強い
Strongest imaginable sensation of any kind	想像できる最大限の

## Appendix B

**Table A2:** Ratings of liking/disliking of sweet-tasting foods (muffin, sweet yogurt and banana).

English	Japanese
Neutral	どちらでもない
Like/dislike slightly	どちらかというと好き／嫌い
Like/dislike moderately	やや好き／嫌い
Like/dislike very much	とても好き／嫌い
Like/dislike very extremely	極めて好き／嫌い
Strongest liking/disliking imaginable	想像できる最大限の好き／嫌い

## Appendix C

**Table A3:** Questionnaire with ratings of appetite, satisfaction and desire for specific tastes (English version).

I am not hungry at all	How hungry do you feel?	I have never been more hungry

Not full at all	How full do you feel?	Totally full

Nothing at all	How much do you think you can eat?	A lot

I am completely empty	How satisfied do you feel?	I cannot eat another bite

Yes, very much	Would you like to eat something sweet?	No, not at all

Yes, very much	Would you like to eat something sour?	No, not at all

Yes, very much	Would you like to eat something salty?	No, not at all

**Table A4:** Questionnaire with ratings of appetite, satisfaction and desire for specific tastes (Japanese version).

まったく 空腹ではありません	どのくらい空腹ですか？	これ以上ないほど 空腹です

まったく 満腹ではありません	どのくらい満腹ですか？	これ以上ないほど 満腹です

まったく 食べられません	今、食事をとるとすると どのくらい食べられると思いますか？	いくらでも 食べられます

まったく満足ではありません	食事や食べ物に関して どのくらい満足感がありますか？	これ以上ないほど 満足です

まったく食べたくない	どのくらい甘いものが食べたいですか？	とても食べたい
	P	
まったく食べたくない	どのくらい酸っぱいものが食べたいですか？	とても食べたい

まったく食べたくない	どのくらいしょっぱいものが食べたいですか？	とても食べたい
